# The international view of envenoming in Brazil: myths and realities

**DOI:** 10.1186/1678-9199-19-29

**Published:** 2013-11-11

**Authors:** Rosany Bochner

**Affiliations:** 1 Institute for Communication and Scientific and Technological Information on Health (ICICT), Oswaldo Cruz Foundation (Fiocruz), Av. Brasil, 4365 - Pavilhão Haity Moussatché, sala 206 - 21045-960 Rio de Janeiro, RJ, Brasil

**Keywords:** Incidence, Snakebites, Scorpionism, Epidemiology, Information systems

## Abstract

Being distant from Brazil’s great natural diversity, from its long tradition in the study of snakebites and from the fact that it is one of the few countries which has a national information system for monitoring incidents involving venomous animals, non-Brazilian researchers face risks when estimating the incidence of these accidents in the country. The present work offers a critical review of the main estimates undertaken since 1954. It is interesting to note contradictions between textual and graphic information within the same article, variations over time in the work of a same researcher and differences among distinct authors, and that all these issues remain unmentioned or undiscussed. Comparison among such estimates and the data available at the Brazilian Information System on Diseases of Compulsory Declaration (Sistema de Informação de Agravos de Notificação – SINAN) creates an opportunity to identify the degree of imprecision present in those articles, and draws attention to the need for the production of studies at both the regional and national levels, based on concrete data collected at national, state and municipal levels, which has been available on the internet since 2001.

## Introduction

Brazil is a country of continental dimensions, encompassing 515,767,049 km^2^ divided into five regions, each presenting geographic, environmental, socioeconomic, cultural and political variations. Given this diversity, it is not difficult to understand the statement of some researchers: “Brazil is a country without standards”. How can one combine data from the extremes of Oiapoque in the north with data from Chuí in the south? How to mix data from Bahia with that from São Paulo? Regardless of the envenoming case studied, one cannot jump into calculations without taking into account the characteristics and diversity of this immense country.

The situation is even more serious when the object of study also depends on the distribution of animals throughout the length of the country, as is the case with accidents involving venomous animals.

Non-Brazilian researchers, then, being unaware of all this complexity, may be taking a risk in estimating the prevalence of envenomings by animals for Brazil as a whole, based solely on data from one or more states or even from a few municipalities. The results are impressive.

## Review

### Snakebite

The first estimate of cases of, and deaths from, snakebite in the world was undertaken in 1954 by Swaroop and Grab [1]. They presented a range between 30,000 and 40,000 annual deaths globally (without including the Soviet Bloc, China or the countries of Central Europe). Within this total, the largest figures were for Asia (25,000 to 35,000) and South America (3,000 to 4,000). For Africa, the authors explained that calculation was difficult and ended up estimating the number of deaths as between 400 and 1,000. In regard to the total number of global cases, they admitted that estimation was difficult, but hazarded an initial guess of around half a million people annually bitten by both poisonous and non-poisonous snakes.

**Figure 1:**
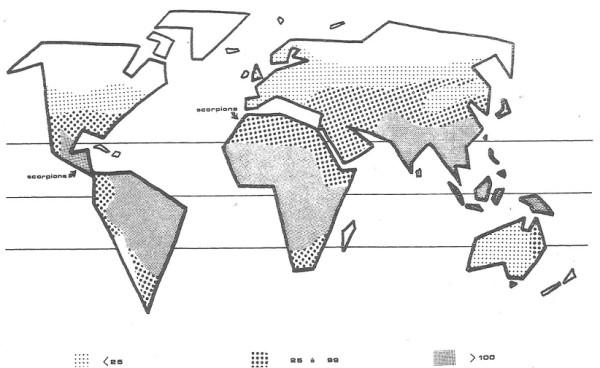
**Snakebite morbidity per 100,000 inhabitants.** Reprinted from “Les complications locales des morsures de serpents” by J. P. Chippaux, *Médecine Tropicale*, 1982, 42(2), 177–183 [2]. Copyright by J. P. Chippaux and *Médecine Tropicale*. Reprinted with permission.

In 1982, a study stated that the worldwide total number of snakebites was difficult to estimate [2]. Nonetheless, the author predicted around 4 to 5 million accidents, and probably more than 150,000 deaths. Such predictions were made based on previous research [3-6]. The study provided a map with snakebite morbidity per 100,000 inhabitants (shown in Figure 1) and informed that its source was a work of the World Health Organization [7]. When this reference was analyzed, neither this map was found nor the snakebite morbidity represented there.

In 1998, Chippaux [8] challenged Swaroop and Grab’s [1] figures, labeling them severe underestimations. According to him, the true incidence of snake envenoming in the world is in excess of five million cases annually, with 125,000 deaths [8]. In 2000, White [9] estimated at more than three million the envenomings by snakes per year, leading to more than 150,000 deaths. In regard to Brazil, Chippaux [8] suggested an incidence between 350 and 450 per 100,000 (household survey), morbidity of 6.8 to 192 per 100,000 (hospital records), mortality of 0.4 per 100,000 (Southwest region) and of 0.4 to 5 (North region) and lethalness 0.4 to 6.5%. The sources of his data were six articles and a summary of a poster, none of which was based on national coverage. Four of the articles presented data from the state of São Paulo [10-13], one contained data from the state of Minas Gerais [14], the last used figures from the state of Acre [15] and the poster presented data from 24 municipalities in the state of Amazonas [16].

The limited number of country-wide studies was pointed out by Bochner and Struchiner [17], when they identified that only four were published in the 20th century: two in 1989 considered to be grey literature [18,19], one in 1993 [20] and one in 1998 [21]. This last was published by the Brazilian Ministry of Health and consists of a manual for the diagnosis and treatment of attacks by venomous animals. It also contains national data for the years 1990 to 1993, despite having been published in 1998 and republished in 2001 [22]. According to this manual, from January 1990 to December 1993 there were a total of 81,611 reported snakebite incidents, which represents an average of 20,000 cases per year for the country. The national rates per 100,000 inhabitants for the period from 1990 to 1993 were 13.78, 13.30, 14.08 and 13.94, respectively. The highest observed rates in this same period were found in the Center-West region: 34.75, 28.36, 37.98 and 32.13, respectively. As can be seen, the official national figures contradict the estimates offered by the aforementioned study [8].

**Figure 2:**
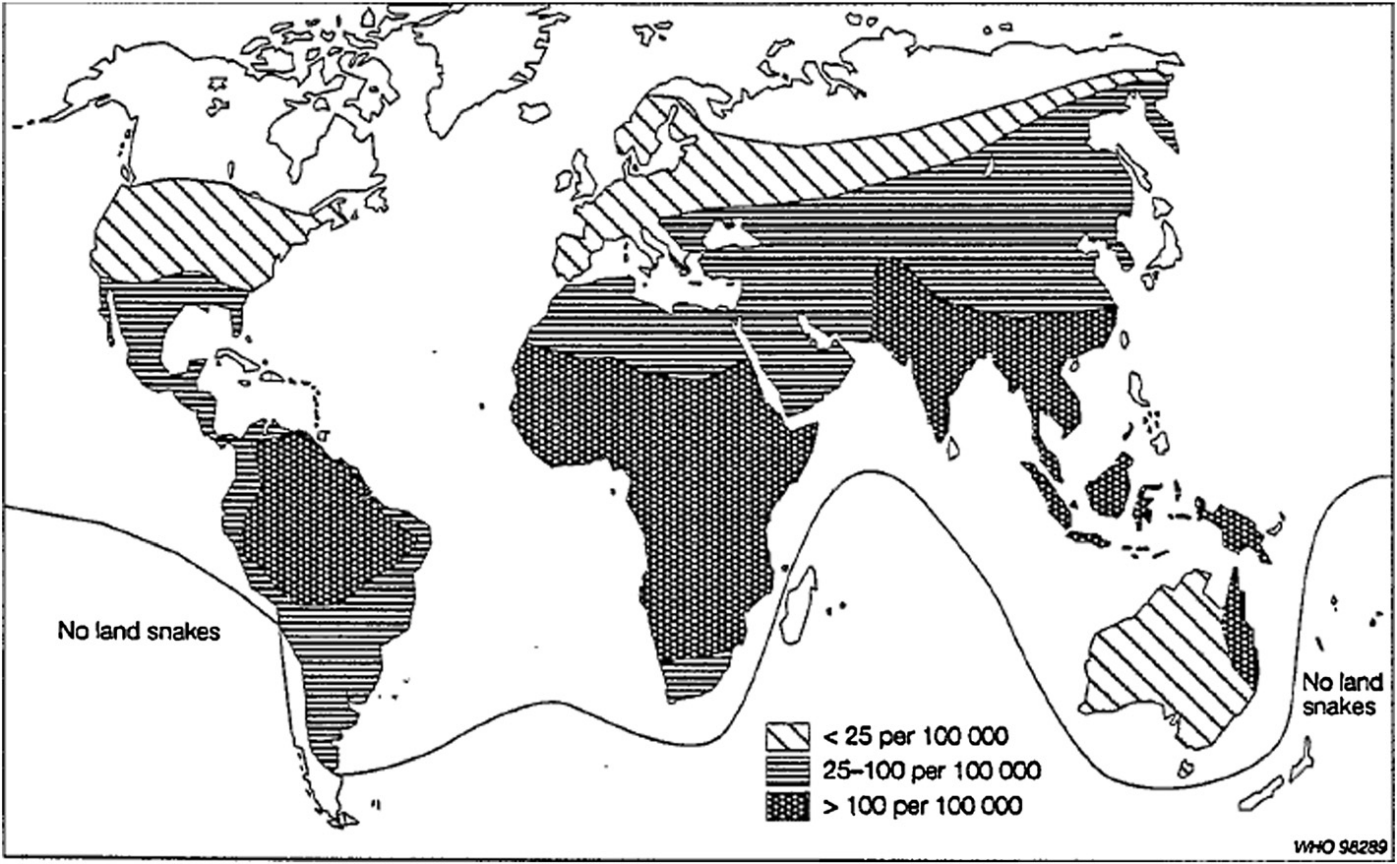
**Map showing the global distribution of snakebite morbidity.** Reprinted from “Snake-bites: appraisal of the global situation” by J. P. Chippaux, *Bulletin of the World Health Organization*, 1998, 76(5), 515–24 [8]. Copyright by WHO.

The same article – based on two chapters of the same book [23,24] and two articles [25,26] – also estimated the incidence of snakebites in Central and South America (with an estimated population of 400 million) as at least 300,000 cases with a total of 150,000 recorded every year, of which 65% receive hospital treatment [8]. According to that study, the number of deaths from snakebite in the entire world exceeds 5,000 and their distribution is probably uneven. Figure 2 reproduces a map that displays the global distribution of snakebite morbidity which was excerpted from that work [8]. It is of interest to observe that the data presented in this graphic do not correspond with the data contained in the same article in reference to Central and South America, as well as for Brazil as a whole [8].

In a study published in 2002, the same estimates of 1998 for Central and South America were maintained: 300,000 bites, of which close to 150,000 were envenomings, most of which were treated in hospitals [8,27].

**Figure 3:**
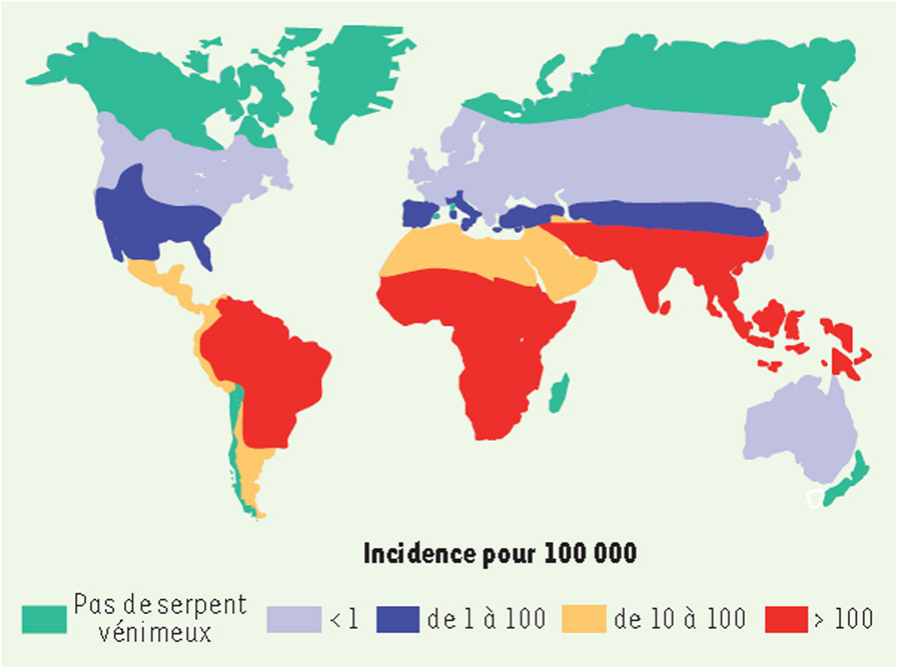
**Annual incidence of snakebites in the world.** Reprinted from “Incidence et mortalité par animaux venimeux dans les pays tropicaux” by Jean-Philippe Chippaux, *Médecine Tropicale*, 2008, 68(4), 334–39 [29]. Copyright by J. P. Chippaux and *Médecine Tropicale*. Reprinted with permission.

A similar map with the global incidence of snakebite envenomations per 100,000 inhabitants was published by Chippaux and Goyffon [28] in 2006 and by Chippaux in 2008 [29,30] and 2009 [31]. The map is reproduced in Figure 3, in which Brazil is categorized using the same color as the majority of African countries. The result is that Brazil is presented as having the same incidence of the continent in which the snakebite rate is more than 100 per 100,000 inhabitants. Despite the fact that the map appears in four articles, only Chippaux [29] offers any indication of the reference bases used to generate the estimates, although with no explanation of how this was done [28-31].

The author states that, in South America, the annual incidence is around 15 to 45 per 100,000 inhabitants, and this, therefore is based on Chippaux [8], Benítez *et al*. [32], Ribeiro *et al*. [13] and Silva *et al*. [33]. However, in Figure 3 the map indicates a rate in excess of 100 per 100,000 inhabitants for the greater part of South America, thus contradicting the statements contained in accompanying text. For the state of Amazônia, the incidence is understood to be between 350 and 590 snakebites per 100,000 inhabitants, with a mortality rate that may reach five per 1000,000 inhabitants. These results were based on Chippaux *et al*. [34], Kerrigan [35] and Pierini *et al*. [15]. It is important to emphasize that, among the group of seven articles cited by Chippaux [29] on which his estimates were based, three refer to data from Brazil, one being from the state of São Paulo [13], one from a small region of the state of Minas Gerais [33] and one from the state of Acre [15]. Such data are limited to generate a solid estimate of the national incidence of snakebites.

The high discrepancy in the incidence attributed to Brazil in Figure 3 may be seen when the value (> 100/100,000) is compared to the 13.8 cases per 100,000 for the country as a whole, and the 52.6 per 100,000 for the North region, where the greatest incidence is observed. These data were published in 2009 by the Ministry of Health in its guide for epidemiological surveillance [36].

In 2008 Kasturiratne *et al*. [37] commented on the work of Swaroop and Grab [1], Chippaux [8] and White [9]. They observed that the study by Swaroop and Grab [1] was based mainly on hospital data, and for this reason its figures on mortality were underestimated, while the works of Chippaux [8] and White [9] did not informed the methodology used to find their estimates. Given that, Kasturiratne *et al*. [37] justified a new estimate of the global burden of snakebite with the application of a more rigorous and repeatable methodology. They grouped 227 countries into 21 geographical regions, following the classification used for the Global Burden of Disease (GBD) project [38] in 2005.

These authors used three strategies to gather primary data: electronic searching for publications about snakebites, selection of specific data on mortality in the relevant countries from databases maintained by United Nations organizations, and identification of grey literature from discussions with key informants. In their results they presented an estimate of maximum and minimum for the number of cases and for the incidence of snakebite per 100,000 inhabitants. For Brazil, which makes up almost exclusively the classification “Latin America, Tropical”, the number of snakebites for the year 2007 was estimated as being between 29,636 and 31,895. The incidence per 100,000 was in the range from 14.97 to 16.12, these values being very different from those presented in Figure 3[28-31].

Kasturiratne *et al*. [37] also estimated for the same region “Latin America, Tropical” the number of deaths as being between 100 and 299, and the mortality rate was given as ranging from 0.051 to 0.151 per 100,000 people. Amongst the sources used by these authors to generate their estimates there are only one article and a book chapter that contain data about Brazil [23,39]. The book chapter is more comprehensive, since it deals with the clinical toxicology of snakebite incidents in South America. The article, on the other hand, has a restricted focus, dealing with snakebites in the urban area of Cuiabá. Judging by the research methodology employed, it apperars that Portuguese-language publications were not included, which might explain the lack of national and regional studies that could minimally represent each region of Brazil. It should be noted, however, that the study of Carvalho and Nogueira [39] is in Portuguese.

**Figure 4:**
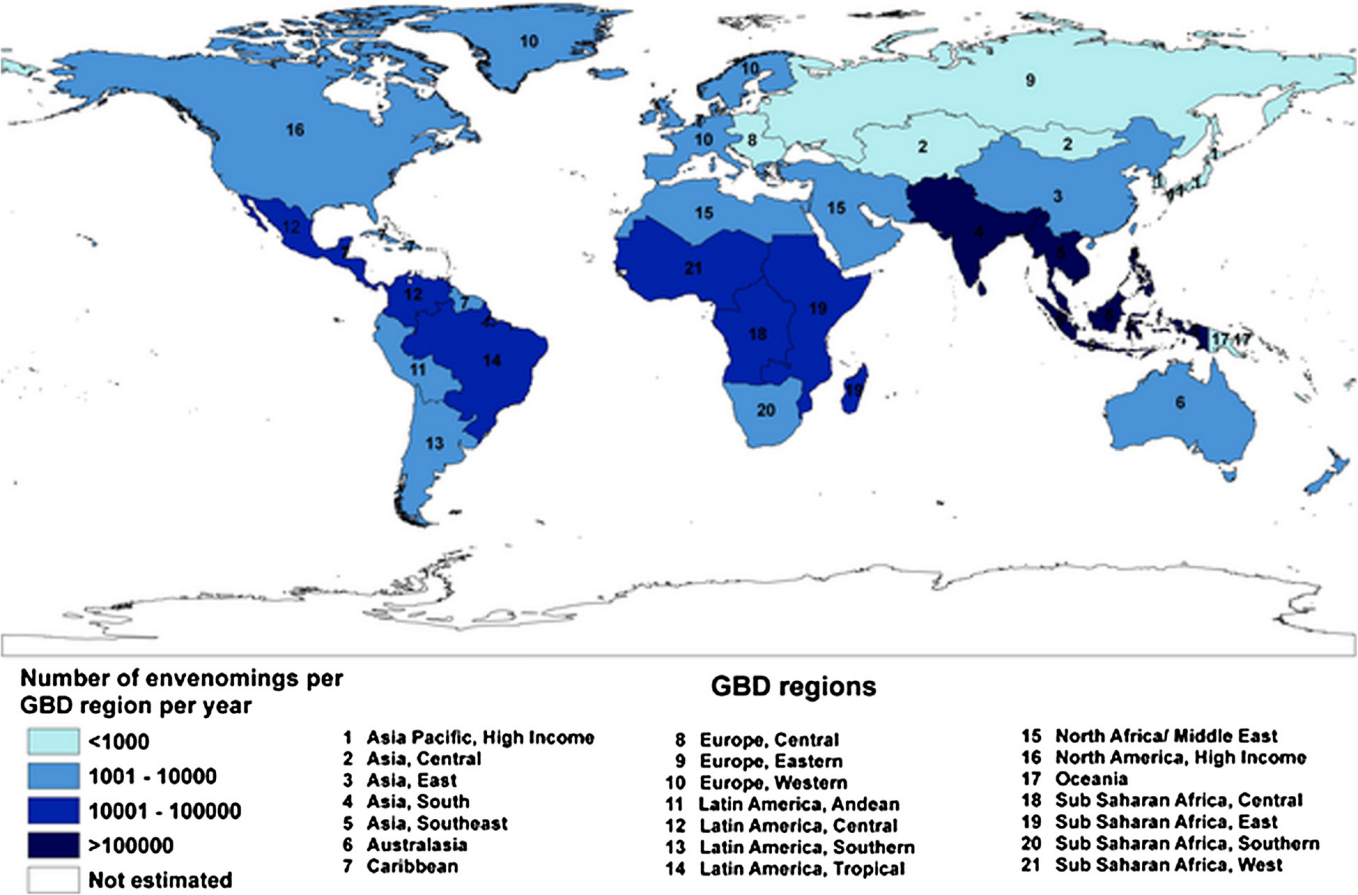
**Regional estimates of envenomings due to snakebite (low estimate).** Reprinted from “The global burden of snakebite: a literature analysis and modeling based on regional estimates of envenoming and deaths” by A. Kasturiratne *et al*., *PLOS Medicine*, 2008, 5(11), e218 [37]. Creative Commons Attribution License (CCAL).

**Figure 5:**
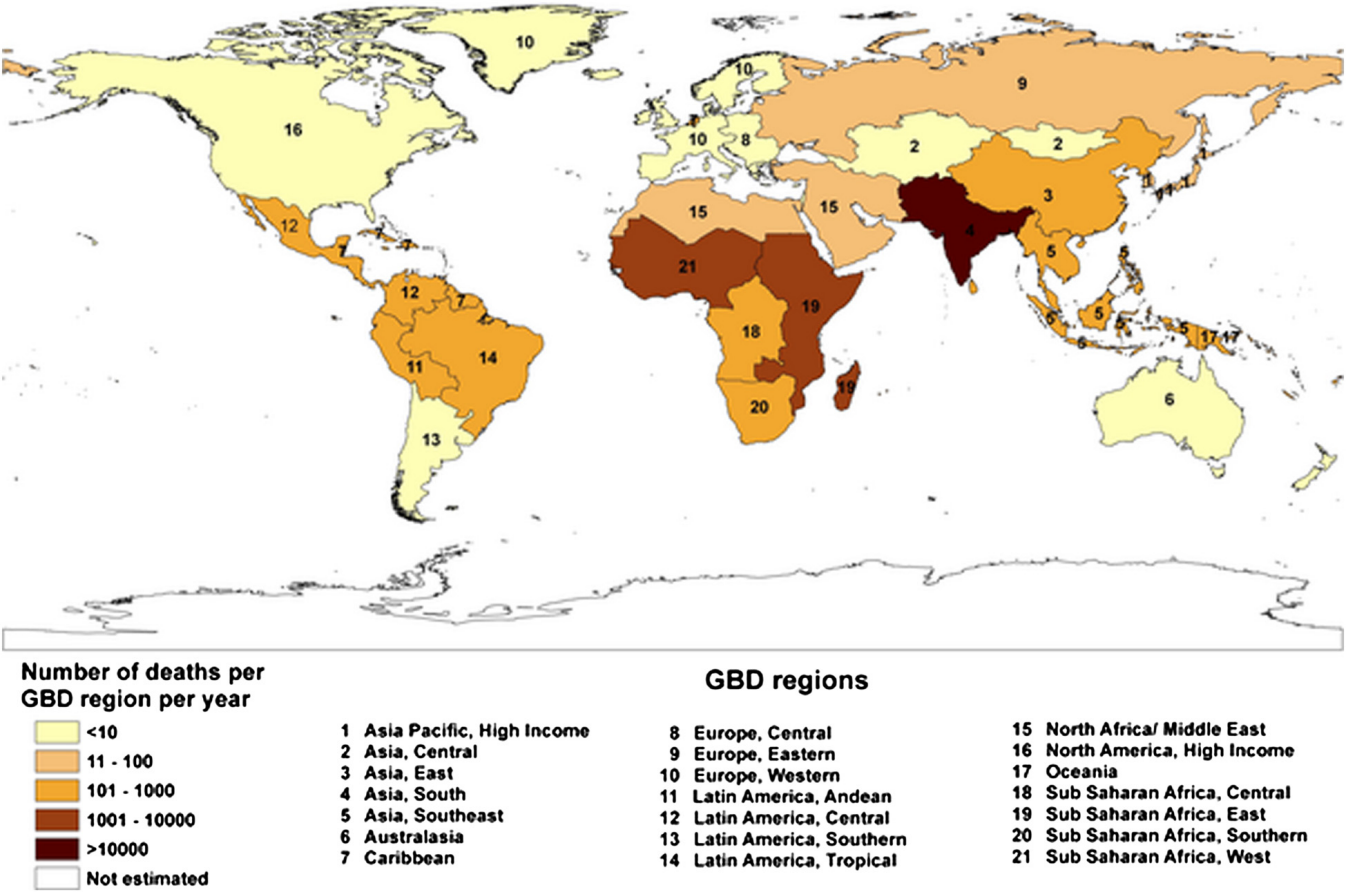
**Regional estimates of deaths due to snakebite (low estimate).** Reprinted from “The global burden of snakebite: a literature analysis and modeling based on regional estimates of envenoming and deaths” by A. Kasturiratne *et al*., *PLOS Medicine*, 2008, 5(11), e218 [37]. CCAL.

The maps that Kasturiratne *et al*. present [37] display minimum values of cases and deaths for snakebites. These are reproduced in Figures 4 and 5, respectively. By not taking into account country populations, and consequently being able to calculate the number of incidents per 100,000 inhabitants, the possibility of undertaking comparative analyses among regions and the various estimates becomes more difficult. According to these maps, Brazil is classified into the category that encompasses 10,001 to 100,000 cases and 101 to 1,000 deaths, figures which are much less clarifying than those presented earlier, and which are derived from the authors’ very own tables [37]. This being the case, the relevance and pertinence of these maps should be questioned, since they do not faithfully represent the data presented in the same article.

In 2010 Warrell [40] analyzed the work Swaroop and Grab [1], Chippaux [8] and Kasturiratne *et al*. [37] and considered them incomplete, faulty, or lacking information about their data-gathering methods, which undermines their extrapolations. He thought that the global estimate of deaths produced by Swaroop and Grab [1] were low, as they were based solely on hospital data and did not took into account the Soviet Bloc, China or the countries of Central Europe. According to Warrel [40], Chippaux’s work [8] extrapolated individual cases that occurred in specific locations within countries when estimating annual global cases. The study carried out by Kasturiratne *et al*. [37] was considered as lacking the essential heterogeneity of the incidence of snakebites within and between countries, and as generalizing this incidence across adjacent territories, thus producing unexpected results (the Caribbean and West Pacific Islands), given that its annual estimates were overly broad.

In 2008 Chippaux [41] had already presented an evaluation similar to that of Warrel [40]. The study suggested that the estimates of Swaroop and Grab [1] were low because of the lack of relevant information and the work of Kasturiratne *et al*. [37] had limitations attributable to its sources and uncertainties about the primary data, which contributed to the creation of overly wide ranges. It also considered that although another research [8], based on a greater number of publications, was more reliable; it presented gaps in information, including the issue of representativeness of local studies within the general epidemiological field.

It is important to point out that Brazil, in contrast to various other countries, possesses national information systems designed to record incidents involving venomous animals. The most important of these is the System on Diseases of Compulsory Declaration (Sistema de Informação de Agravos de Notificação – SINAN), as it has a specific module for recording that type of incident, which makes the information more detailed and appropriate for epidemiological analysis of this health issue [42,43].

In the face of all these estimates made by non-Brazilian researchers, the following question arises: what does SINAN have to offer with respect to these figures? By way of comparison, the data on snakebites recorded by SINAN for the year 2007 were gathered. The system recorded 27,030 cases and 125 deaths, suggesting an incidence of 14.28 per 100,000 inhabitants and a mortality rate of 0.066 [44].

It can be noted that the estimates of minimum and maximum figures given by Kasturiratne *et al*. [37] for the year 2007 contain the values indicated by SINAN for cases, deaths, incidence and mortality rate, despite the fact that the maps displayed values that widely differed from the reality in Brazil. Once again, the relevance and pertinence of these maps should be questioned.

In 2008 Bochner and Fiszon [45], two Brazilian researchers, using the data from SINAN for the period from 2001 to 2006, developed a profile of incidents involving venomous animals in Brazil, taking into account regional variations. In that study, snakebite morbidity for the country was defined as 14 per 100,000 inhabitants, with the highest observed value being in the North region, at 49 per 100,000 inhabitants.

In 2010, a group of four researchers, formed by three non-Brazilians and one Brazilian, published an article in which they indicated that, in Brazil, health statistics related to venomous animals were satisfactory [46]. This group mentioned the work of Oliveira *et al*. [47], which covers the epidemiology of animal envenoming in Brazil, based on data collected by the Ministry of Health by means of SINAN from 2000 to 2007.

### Scorpionism

**Figure 6:**
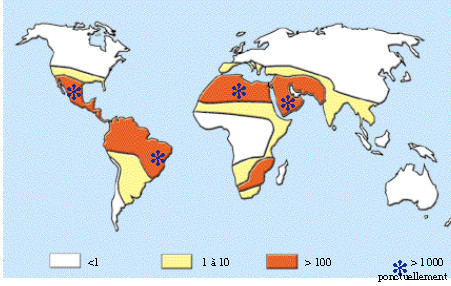
**Incidence of scorpionism in the world.** Reprinted from “Envenimations et intoxications par les animaux venimeux ou vénéneux. I. Généralités” by J. P. Chippaux and M. Goyffon, *Médecine Tropicale*, 2006, 66(3), 215–220 [28]. Copyright by J. P. Chippaux and *Médecine Tropicale*. Reprinted with permission.

Chippaux and Goyffon [28] suggested that scorpions are found abundantly in tropical regions, especially in humid locations of hot subdesert zones. So, they showed that the morbidity and mortality from scorpion bites were particularly higher in Central America, North Africa and the Middle East. Their work included a map showing the global incidence of scorpion envenoming, reproduced in Figure 6. How such estimates were obtained is not informed, and the references did not include any article dealing with scorpionism in Brazil.

The map presented in Figure 6 contains a few mistakes, the most general of which is the fact that there is no way to represent any incidence rate greater than 10 and less than, or equal to, 100. In the case of Brazil, the SINAN data from 2006 shows that the North region of the country does not have the same incidence as the Northeast. Furthermore, even the Northeast, with the highest national incidence, does not exceed the figure of 100. Neither of the states in this region, Pernambuco or Alagoas, shows an incidence over 100, thus leaving hanging the question of where exactly is the location whose incidence exceeds 1,000, as indicated on the map.

**Figure 7:**
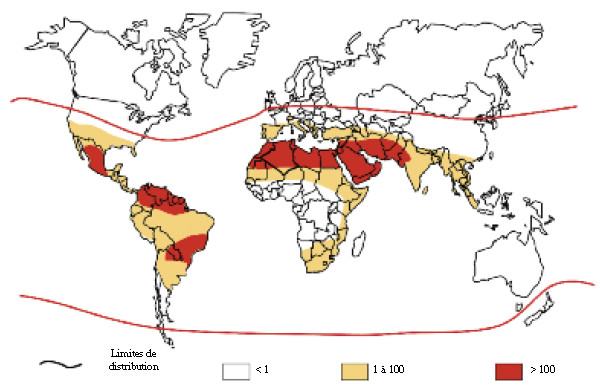
**Annual incidence of scorpionism in the world (per 100,000 inhabitants).** Reprinted from “Incidence et mortalité par animaux venimeux dans les pays tropicaux” by J. P. Chippaux, *Médecine Tropicale*, 2008, 68(4), 334–39 [29]. Copyright by J. P. Chippaux and *Médecine Tropicale*. Reprinted with permission.

In 2008 Chippaux and Goyffon [48] reviewed the literature of the last 30 years in order to discuss the global epidemiology of scorpion bites. They published a map showing the annual incidence of scorpion bites per 100,000 inhabitants, which is reproduced in Figure 7. This same map was also used in three articles by Chippaux [29-31] published in 2008 and 2009, as well as in his presentation delivered in 2013 at the 1st International Conference Vital for Brasil [49]. It is interesting to observe how the estimates for Brazil changed by comparing Figures 6 and 7 and, although the authors are practically the same, there is no mention regarding these changes. Figure 7, on the other hand, has remained the same since 2008, which is puzzling, given the changes which have taken place in Brazil with regard to increased populations of both humans and scorpions.

According to Figure 7, the highest rates of scorpion stings in Brazil are found in all the states of the Southeast and South regions, and part of the North (Roraima, Amapá and parts of Amazonas and Pará), the Northeast (a small area in Bahia) and Center-West (Mato Grosso do Sul, Goiás and part of Mato Grosso). The data from SINAN offer a different picture of the distribution of scorpion stings in Brazil. The rate does not exceed 100 per 100,000 in any state. The region with the highest rate is the Northeast, followed by the Southwest, North, Center-West, and finally by the South [44,45].

**Figure 8:**
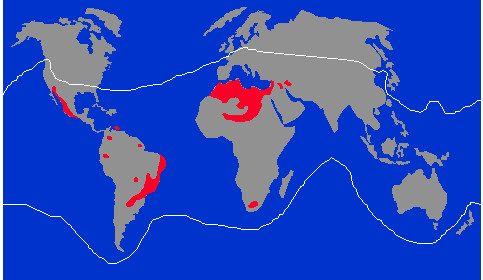
**Worldwide scorpion distribution (white solid line).** In red, areas with severe problems of scorpionism. Reprinted from “The evolution of scorpionism in Brazil in recent years” by W. R. Lourenço *et al*., *The Journal of Venomous Animals and Toxins*, 1996; 2(2): 121–134 [58]. Copyright by CEVAP/UNESP. Reprinted with permission.

**Figure 9:**
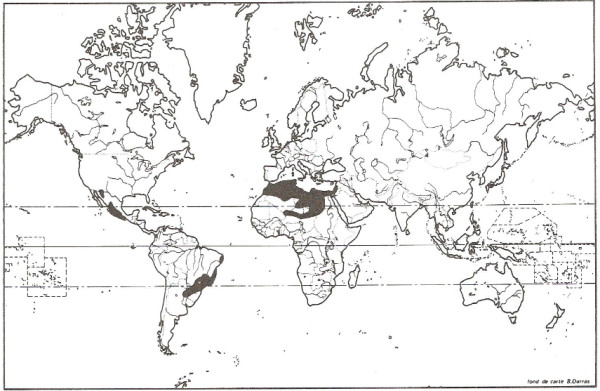
**Areas with high rates of scorpionism worldwide.** Reprinted from “Peut-on parler d’une biogéographie du scorpionisme” by W. R. Lourenço, *Compte*-*Rendu des Séances de la Société de Biogéographie*, 1988, 64(4), 137–143 [59]. Copyright by W. R. Lourenço and Société de Biogéographie. Reprinted with permission.

Perusal of the articles used by Chippaux and Goyffon [48] as the sources for the estimates presented in the map reveals nine studies: three with data from the state of Bahia [50-52], two with data from the state of São Paulo [53,54], two with data from the state of Minas Gerais [55,56], one with data from the state of Pará [57] and one with data from the country as a whole [58]. The last one, published in 1996, contains a map showing the distribution of scorpions across the world and those regions which have severe scorpion envenoming problems. This map is reproduced in Figure 8. It is interesting to observe that in 1988 Lourenço [59] had already published a similar map, shown in Figure 9, in which only the three largest areas in red from Figure 8 are represented as having high rates of scorpion stings.

It can be seen that part of the Brazilian areas marked in red in Figure 8 do not correspond to the areas of high incidence of scorpion stings presented in Figure 7.

## Conclusions

The estimates of the incidence of snake and scorpion envenoming in Brazil made by foreigners have shown themselves to be unsatisfactory and not a faithful reflection of the reality in the country.

Very often, due to the fact that data are presented in maps, the discrepancies have gone unnoticed and have been reproduced over time in various publications.

The methodology of developing indicators based on different regional studies is highly sensitive to the choice of works to be included in the sample. In many cases the estimates are based on very heterogeneous data collected from highly specific locations, which generates unrepresentative and untrustworthy information. It is thus very necessary to be familiar with the peculiarities of Brazil, which is not an easy task, even for a native.

Brazil has a long tradition in the production, control and distribution of antivenom serum, as well as in the free treatment of victims. A concern in regard to information about envenoming has been present in Brazil ever since the first ampoule of serum was delivered to the populace by Vital Brazil in 1901 [60].

Currently, the duty of reporting poisoning occurrences has changed from being obligatory to being compulsory [61]. The records of these reports are the basis of the Information System on Diseases of Compulsory Declaration (SINAN), and, despite the possibility of underreporting in some geographic areas; these records constitute the best picture of the Brazilian reality in regard to envenoming. Thus, the best course is to leave estimates to one side and to undertake research at national and regional levels, based on concrete data from the country as a whole, its states and municipalities. Such data have been available on the Internet since 2001.
